# Artificial neural network-based model to predict the effect of γ-aminobutyric acid on salinity and drought responsive morphological traits in pomegranate

**DOI:** 10.1038/s41598-022-21129-z

**Published:** 2022-10-05

**Authors:** Saeedeh Zarbakhsh, Ali Reza Shahsavar

**Affiliations:** Department of Horticultural Science, College of Agriculture, Shiraz University, Shiraz, Iran

**Keywords:** Computational platforms and environments, Image processing, Machine learning, Plant stress responses

## Abstract

Recently, γ-Aminobutyric acid (GABA) has been introduced as a treatment with high physiological activity induction to enhance the ability of plants against drought and salinity stress, which led to a decline in plant growth. Since changes in morphological traits to drought and salinity stress are influenced by multiple factors, advanced computational analysis has great potential for computing nonlinear and multivariate data. In this work, the effect of four input variables including GABA concentration, pomegranate cultivars, days of treatment, and drought and salinity stress evaluated to predict and modeling of morphological traits using artificial neural network (ANN) models including multilayer perceptron (MLP) and radial basis function (RBF). Image processing technique was used to measure the LLI, LWI, and LAI parameters. Among the ANNs applied, the MLP algorithm was chosen as the best model based on the highest accuracy. Furthermore, to predict and estimate the optimal values of input variables for achieving the best morphological parameters, the MLP algorithm was linked to a non-dominated sorting genetic algorithm-II (NSGA-II). Based on the results of MLP-NSGA-II, the best values of crown diameter (18.42 cm), plant height (151.82 cm), leaf length index (5.67 cm), leaf width index (1.76 cm), and leaf area index (13.82 cm) could be achieved with applying 10.57 mM GABA on ‘Atabaki’ cultivar under control (non-stress) condition after 20.8 days. The results of modeling and optimization can be helpful to predict the morphological responses to drought and salinity conditions.

## Introduction

It is well-concluded that plants are always exposed to several abiotic stresses simultaneously or in sequence under field conditions^1,2^. Drought and salinity are the most frequent co-occurring abiotic stresses in natural environments that cause enormous losses in crop productivity worldwide^3^. Both drought and salinity stress significantly impact on many aspects of plant morphology and physiology including the accumulation of reactive oxygen species, the disorderliness in the stomatal conductance, changes in the growth patterns and biomass (morphology of leaves and roots), respiration and photosynthesis rate, water uptake and nutrient balance and as well as induce the osmotic stress and ion toxicity in plants^4,5^. Hence, plant growth, yield, and distribution of plant species are remarkably influenced by drought and salinity stress^6,7^.

In the last decade, the application of many treatments has been comprehensively investigated to mitigate the negative impacts of stressful conditions on plant growth. Besides this, the application of natural products or plant growth regulators (PGRs) as bio-stimulants and organic nature has been popularized due to a cost-effective, safe, and eco-friendly way^8^. γ-Aminobutyric acid (GABA), one of the PGRs with four-carbon non-protein amino acid, is well documented as an endogenous plant metabolite or novel signaling molecule that can rapidly accumulate in a plant cell under stressful conditions and become involved in physio-biochemical functions for surviving the plants^9,10^. Therefore, the great majority of studies have pronounced that the exogenous application of GABA has a key role to mitigate the undesirable effects of various abiotic stresses in different plants such as prunus (*Prunus avium*)^11^, pepper^12^ and tea (*Camellia sinensis*)^13^. It is important to highlight that a very low level of endogenous GABA produces in plants and subsequently, the exogenous treatment of GABA leads to an increase in the level of internal GABA under both environmental and normal conditions^10^. Activation of enzymatic, and non-antioxidant defense system, maintaining the balance of carbon to nitrogen ratio, regulating the plant growth and development, involving in the carbohydrate and amino acid metabolism, enhancing the morphological growth and function of the photosynthetic machinery, chlorophyll biosynthesis and membrane stabilization, osmoregulation, pH change, and glutamate homeostasis are the important candidate-roles by boons of exogenous GABA in plant under stressful condition such as drought and salinity^14,15^.

Iran has the first rank in the world in terms of production, variety of cultivars, and quality of pomegranate. In recent years, severe droughts, soil salinity, and reduction in groundwater resources in Iran have been the most important factors reducing appropriate pomegranate yield^16^. Besides this, the leaf area and growth of the pomegranate plant as crucial parameters are affected by water and salinity stress^6^. Therefore, it is crucial to improve the drought and salt tolerance of pomegranate through an economical and effective strategy in the large-scale area. Measurement of leaf morphological traits by conventional methods is time-consuming and has low accuracy. Also, human error reduces the accuracy of the measurement. Hence, image processing as a novel, reliable, simple, high-speed, and cost-effective machine vision method offers greater detailed information from the morphological properties^17^. Image processing can be a useful method to evaluate and measure leaf morphology using indexes derived from RGB (red–green–blue) images to reduce the errors caused by human bias and the conventional methods.

Since the effect of different concentrations of GABA, genotypes, salinity and drought stress on plant morphology can be considered as a multivariable process; however, finding the association between several morphology traits requires dealing with uneven and nonlinear datasets which have been generated from the multivariable process. Nowadays, artificial intelligence models such as neural networks and genetic algorithms as advanced computational analysis are reliable prediction models for the analysis of large, multi-dimensional, uneven, and nonlinear datasets^18^. Considering the information processing and decision-making capabilities of artificial neural networks (ANNs), this technology is similar to the structure of the human neural network. Statistical ANN has been extensively used in different fields of science including plant sciences^19^, environmental sciences^20^, remote sensing^21^, and engineering^22^. Generalized regression neural network (GRNN), multi-layer perceptron (MLP), multi-layer network with radial basis functions (RBF), and probabilistic neural network (PNN) are the different types of ANNs^23^. One of the advantages of ANN is that it does not require any previous knowledge concerning the inter-relationships among input and output variables^24^. MLP neural network model as one of the most well-known neural network algorithms, like other ANNs, is made up of a large number of neurons, each neuron with its weight^25^. In other words, in the hidden layers, the number of neurons plays a significant role in the MLP's design. RBF, like the MLP, is another kind of statistical ANNs, that has the same functionality as MLP but is effective for use in more than one dimension. Wherever appropriate characteristics are included, RBF is claimed to be successful for predictions that use approximation multivariate functions^18^. However, neural network models have good learning capabilities, while they cannot interpret results^26^. In this regard, optimization techniques can help in interpreting the results. In general, there are two categories of optimization techniques including classical and evolutionary or metaheuristic algorithms^27^. Evolutionary algorithms are the most widely used methods, since regardless of the type of problem in terms of being linear or nonlinear quickly converge to the globally optimal solution (close to optimal)^28^. Recently, the Genetic Algorithm (GA) as one of the most well-known evolutionary optimization algorithms is widely used for optimizing many important problems. However, as a single objective optimization algorithm, it can only find the optimal level of inputs for each target variable separately, not for all target variables simultaneously^29^. Therefore, non-dominated sorting genetic algorithm-II (NSGA-II) as an evolutionary multi-objective optimization algorithm that is useful for finding the dominant responses through the Pareto front in a multi-objective function may be preferable. This searching algorithm was introduced by Deb et al.^30,31^ and inspired from natural selection and survival of the fittest. Furthermore, in large datasets, ANN-NSGA-II is an efficient tool for predicting and interpreting significant factors that cause an improvement of a specific result^32^. In other words, the combination of ANNs with NSGA-II is able to reduce computational volumes and obtain simultaneously the best combination of inputs to improve a set of optimal responses for a specific problem^33^. Recently, the efficiency of ANN-NSGA-II as an optimized algorithm for predicting and finding the best morphological traits of citrus species under drought stress has been demonstrated^19^. They reported that the constructed ANN-NSGA-II as a reliable computational tool had good efficiency in predicting citrus morphological responses to drought stress. However, there is a lack of a fundamental study on the effectiveness of the ANN-NSGA-II for modeling and optimizing plant morphology against the combination of drought and salinity stress.

Although the successful effect of GABA has been reported to protect different plants under different abiotic stresses; however, the protective effect of GABA under salinity-drought stress remains unknown. Therefore, the aim of the present study was: (i) investigate the role of exogenous GABA on morphological parameters under drought, salinity and their combination in two commercial cultivars of pomegranate (*Punica granatum* cv. Rabab and Atabaki), (ii) measuring the leaf dimensions by indexes derived from RGB as an appropriate method, (iii) using and comparing two well-known ANNs model including multilayer perceptron (MLP) and radial basis function (RBF) for predicting morphological parameters under mentioned conditions and link the best ANN model with NSGA-II to achieve optimal morphological parameters by optimal condition.

## Materials and methods

### Plant materials and experimental design

Two cultivars of 2-year-old pomegranate (*Punica granatum* cv. ‘Rabab’ and ‘Atabaki’) were obtained from a commercial nursery and immediately transplanted into 10 L black plastic pots filled with a mixture of soil + leaf litter (3:2 w/w). Both cultivars were kept in the greenhouse (28 ± 1 °C, 60 ± 5% relative humidity, L16:D8 h photoperiod) at the College of Agriculture, Shiraz University, Iran. Plants were fertilized once a week using a half-strength Hoagland solution for 4 months until the beginning of the experiment. In June 2021, plants were exposed to drought stress (100% and 60% of field capacity (FC), salt stress (0 and 60 mM of NaCl), and drought stress plus salt stress (60% FC + 60 mM of NaCl) for 45 days. For the GABA treatment, pomegranate leaves were sprayed with (0, 10, 20, and 40 mM) GABA (Sigma Aldrich, St. Louis, MO, USA) three times at 15 days intervals and immediately exposed to abiotic stress. This experiment was conducted as a completely randomized design (CRD) with a factorial arrangement and 4 replicates.

### Morphological evaluations

Growth and morphological characteristics including plant height (PH), crown diameter (CD), leaf length index (LLI), leaf width index (LWI), and leaf area index (LAI) were measured based on centimeter units before the beginning of stress treatments and after 14 days, 30 days and 45 days from the beginning of stress, respectively.

**Figure 1:**
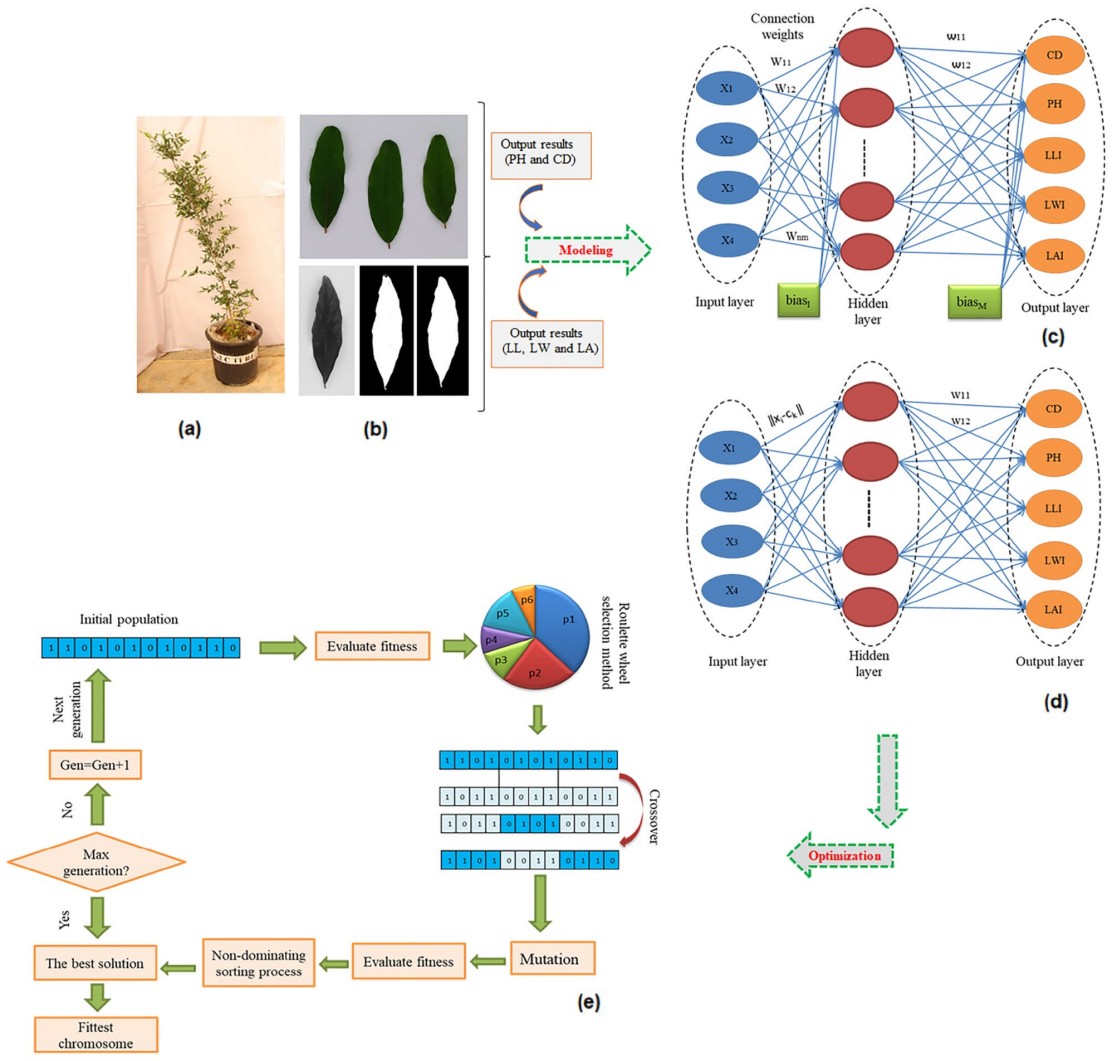
Schematic diagram of the procedure used in this study (**a**,**b**) image processing and morphological measurements, (**c**,**d**) modeling morphological traits based on four input variables including pomegranate cultivars, GABA concentrations, stress treatments and days of treatment using multilayer perceptron (MLP) and radial basis functions (RBF), respectively, and (**e**) the step-by-step optimization process of morphological traits via non-dominated sorting genetic algorithm-II (NSGA-II).

The CD and PH were measured based on manual measurements (Fig. 1a) and the image processing method was carried out to collect data of the LLI, LWI, and LAI traits from mature leaves of similar age. Images were captured using a smartphone camera (Huawei Y6 Prime 2019 MRD-LX1F with 13-megapixel resolution and auto-focus feature). Based on previous studies, smartphone-based imaging is a reliable method for studying the morphological traits of plants^32,34^. Leaf images were captured from a vertical distance of 14 cm of the samples in the lightbox with white background as an image acquisition environment. White-colored LED lamps (2000 Lux) were used as a source of light in the lightbox. The dimensions of the lightbox were 20 cm × 33 cm. Image processing-based measurements have been carried out with MATLAB^35^ software (MathWorks, Inc., Natick, USA). The software-based on image processing, such as ImageJ, Macf-IJ, LAMINA, Lamina2-shape, and MATLAB have been proposed to describe plant's phenotype, and leaf dimensions and classify plant leaves^36–38^. However, to measure the leaf dimensions, the MATLAB software often provides outperforms and has higher accuracy than the other mentioned software^39^. The image processing steps were performed as follows: (1) the image segmentation with threshold method to segment the leaf from the background and convert the image to grayscale; (2) components the split color to red, green, and blue color channels and generate a matrix of pixels from the binary image; (3) eliminating noises of binary images and filling holes; (4) recognizing the leaf tip and the petiole of pomegranate; and (5) measuring the image LLI, LWI and LAI (Fig. 1b). Afterward, the LAI was determined based on the measurement of white pixels described by Nguyen^40^. The LWI (pixels) was acquired based on the number of pixels at the widest points of the lamina perpendicular to the midrib and the LLI of the pomegranate (pixels) was determined using the two-point distance from the lamina tip to the petiole intersection along the midrib based on the formula in Eq. (1).

1 $$D = \sqrt {\left( {a_{i} - b_{i} } \right)^{2} - \left( {a_{j} - b_{j} } \right)^{2} }$$

where D represents the distance between points a and b, *a*_*i*_, *b*_*i*_ and *a*_*j*_, *b*_*j*_ are the abscissa and the ordinates of points a and b, respectively.

The LLI and LWI were calculated based on the method of Zhang^39^. Following the obtaining of the LLI, LWI, and LAI in pixels, based on the number of white pixels per centimeter, the pixel values were converted to actual values. Ultimately, measurements of leaf morphological traits were derived from the ratio of the number of pixels on a white background to the actual size. The Pearson correlation between morphological traits was estimated using *corrplot* package^41^ in R software version 3.6.1.

### ANN modeling analysis

In this study, two of the most commonly-known feed-forward ANNs including MLP and RBF were employed to model and predict the morphological traits. Before using ANN modeling, data standardization was performed to improve the efficiency of the model and decrease the mean squared error (MSE). The datasets were standardized by the z-score normalization technique as follows:

2 $${{X}}_{{{i}}}^{^{\prime}} = \frac{{{{X}}_{{{i}}} - \overline{{X}}}}{{{\sigma}}}$$

where $X_{i}^{^{\prime}}$, $X_{i}$ and $\overline{X}$ are standardized values of $X$, the value of the *i*th observation data and mean of observed values, respectively. $\sigma$ is the standard deviation.

In the present study, independent variables including the concentration of GABA, days after applying treatments, pomegranate species, and salinity-drought stress were considered as the input layer and CD, PH, LLI, LWI, and LAI were considered as targets of the ANN model. Like most ANN methods, the MLP and RBF operate through training and testing. To analyze the performance of each model, the dataset was randomly shuffled and divided into the training subset and test subset. The dividing of datasets plays an important role in the performance of ANN. The training and test set was employed to predict the model parameters and to check the generalization ability of the model, respectively. There are no generally accepted rules for determining the size of training data for proper training; however, the training set is representative of the entire input sample set and often requires a substantial amount of all spectra of the input sample set^42^. To training and testing the neural network, 80% and 20% of data lines were randomly chosen, respectively.

### MLP model

The MLP consists of three layers including one input layer, some hidden layers, and one output layer. Figure 1c shows an MLP model with one hidden layer. To train the MLP algorithm, a back-propagation Bayesian algorithm with bias learning function and momentum weight was used. To find the best topology of the MLP algorithm and maximize the accuracy of outputs, various values of hidden layers and neurons in each hidden layer should be determined based on trial and error to improve the overall performance of the optimally constructed model. Because, a large number of hidden layer neurons complicates the network, and a small number of neurons simplifies the network. Therefore, underfitting may happen by too simple a network; conversely, too complex a network can lead to overfitting^28–43^. Hence, the MLP neural network was established based on four layers (an input layer, two hidden layers, and an output layer), and the optimal number of hidden layer neurons was considered fourteen and twelve, respectively. Each neuron unit produces an output based on a hyperbolic tangent sigmoid function (tansig) and linear function (purelin) from hidden layers and output layer, respectively^44^. The error between the input and output of the variables is minimized through the following formula:

3 $$E = \frac{1}{K}\mathop \sum \limits_{k = 1}^{K} \left( {y_{k} - \hat{y}_{k} } \right)^{2}$$

In a three-layer MLP with *n* inputs and *m* neurons in the hidden layer $\hat{y}$ determined as the formula:

4 $$\hat{y} = f\left[ {\mathop \sum \limits_{j = 1}^{m} w_{j} \cdot g\left( {\mathop \sum \limits_{i = 1}^{n} w_{ji} x_{i} + w_{j0} } \right) + w_{0} } \right]$$

where $x_{i}$, $w_{0}$, $w_{j0}$, $f$, $g$, $w_{ji}$, and $w_{j}$ represent the *i*th input variable, the bias related to the neuron of output, the bias of the *j*th the neuron of the hidden layer, the transfer functions for the output layer, the transfer functions for the hidden layer, the weight connecting the *j*th the neuron of the hidden layer and the *i*th input variable, and the weight linking the neuron of output layer and the *j*th the neuron of the hidden layer.

### RBF model

There are only three layers in the RBF: an input layer, a single hidden layer, and an output layer. Network structure RBF is shown in Fig. 1d. The input of the transfer function for each neuron in such a network is the Euclidean distance between the input and the center of that neuron. The RBF-ANN process works as follows: First, input data enter into the network (input layer), then, assesses the similarity between the input data and the prototype stored therein by hidden layer neurons through the Gaussian radial basis function (transfer function) defined in Eq. (5).

5 $$\varphi \left( {\left\| {x_{i} - c_{k} } \right\|} \right) = e^{{ - \left( {\frac{{\left\| {x_{i} - c_{k} } \right\|^{2} }}{{2\sigma_{k}^{2} }}} \right)}}$$

where $\varphi$, $x_{i}$, $c_{k}$, $\sigma_{k}$ and $\left\| {x_{i} - c_{k} } \right\|$ denotes the Gaussian function, the input vector of the neuron in the hidden layer, the center and the width of the *k*th RBF unit and the Euclidean distance norm.

Then, to calculate the RBF output, the outputs of the non-linear Gaussian function are integrated linearly using the weighted average method in the output layer by the following equation:

6 $$y_{i} = \mathop \sum \limits_{j = 1}^{n} \omega_{ji} \varphi_{j} \left( x \right)$$

where $w_{ji}$ denotes the *i*th weight between the hidden layer and output layer, and *n* represents the number of hidden nodes. When the similarity between the input and the prototype is high, the RBF neuron's output is closer to 1, and when it's not, it's close to 0.

### Assessment of statistical model performance

Three statistical models were employed to error estimations and compare the accuracy and performances of ANN models in predicting the target values. The assessment criteria included root mean square error (RMSE), mean bias error (MBE), and coefficient of determination (R^2^) as follows:

7 $$R^{2} = \left. {\left[ {\frac{{\mathop \sum \nolimits_{i = 1}^{n} \left( {yi - \overline{y}} \right)\left( {\hat{y} - \widehat{{\overline{y}}} } \right)}}{{\sqrt {\mathop \sum \nolimits_{i = 1}^{n} \left( {yi - \overline{y}} \right)\sqrt {\mathop \sum \nolimits_{i = 1}^{n} \left( {\hat{y} - \widehat{{\overline{y}}}} \right)} } }}} \right.} \right]^{2}$$

8 $$RMSE = \sqrt {\frac{{\left( {\mathop \sum \nolimits_{i = 1}^{n} \left( {y_{i} - \hat{y}_{i} } \right)^{2} } \right)}}{n}}$$

9 $$MBE = \frac{1}{n}\mathop \sum \limits_{i = 1}^{n} \left( {y_{i} - \hat{y}_{i} } \right)$$

where $y_{i}$ is the value of predicted datasets, $n$ is the number of measured values, $\hat{y}_{i}$ is the mean of measured values and $\overline{y}$ is the mean of measured values.

### Sensitivity analysis

To identify which input variables were more important than the other to reach optimal output variables in the model, sensitivity analysis was evaluated by the following criteria:

- The variable sensitivity error (VSE) value indicates the performance of the ANN model if that input variable is unavailable.
- The value of variable sensitivity ratio (VSR) value indicates the relative ratio between the error of the ANN model and VSE if all input variables are available. Therefore, the more important input variable can be ranked based on higher VSR.

### Optimization of ANN models using NSGA-II (non-dominated sorting genetic algorithm-II)

The trained MLP models were processed as the fitness function using NSGA-II to find the optimal input variables to produce the best values of targets. During the optimization process, the roulette wheel selection method was considered to select an elite population for crossover. Furthermore, to create the next generation of chromosomes, crossover function, and mutation were applied. Mutation can create random variations in chromosomes and reduce the possibility of having similar chromosomes, thus, local minima in the population are decreased^28^. To achieve the best fitness, 2-point crossover was considered with an 80% possibility, 800 was fixed as the number of generations, initial population, and mutation rate was set to 100 and 0.04 respectively (Fig. 1e). The optimal values of the mentioned parameters should be estimated by using trial and error. Also, the fitness function based on the results of the MLP model was formulated (Eq. 10), to maximize CD, PH, LLI, LWI, and LAI traits.

10 $$\normalsize {{F}} = \sqrt {\left( {{{Y}}_{{{{CD}}}} - {{c}}} \right)^{2} + \left( {{{Y}}_{{{{PH}}}} - {{d}}} \right)^{2} + \left( {{{Y}}_{{{{LL}}}} - {{e}}} \right)^{2} + \left( {{{Y}}_{{{{LW}}}} - {{f}}} \right)^{2} + \left( {{{Y}}_{{{{LA}}}} - {{g}}} \right)^{2} }$$

where $Y_{CD}$, $Y_{PH}$, $Y_{{LL{\text{I}}}}$, $Y_{{LW{\text{I}}}}$, $Y_{{LA{\text{I}}}}$ are CD, PH, LLI, LWI, and LAI traits, respectively, and $c$, $d$, $e$, $f,$ and $g$ are the maximum CD, PH, LLI, LWI, and LAI traits, respectively.

All statistical computational for assessing ANNs algorithm and ANN-NSGA model were conducted by MATLAB^35^ software.

### Statement on guidelines

All experimental procedures on pomegranate plants complied with relevant institutional, national, and international guidelines and legislation.

## Results

### Evaluation of pomegranate phenotypes with or without exogenous application of GABA under drought and salt stress

The obtained primary results demonstrated that morphological parameters of pomegranate including CD, PH, LLI, LWI, and LAI were influenced by the negative effects of drought and salinity stresses and their combination (Table S1). Under the mentioned conditions, the negative effect of salinity-drought stress on decreasing the morphological traits was more than of the drought and salinity stress alone. It is noticeable that the stress-treated plants were extremely affected by stressful conditions, especially at the initiation time of stress treatments (15 days); however, a slight decrease in morphological traits at the middle and the end of treatment time (30 and 40 days) were observed. Furthermore, the type of cultivar (‘Atabaki’ and ‘Rabab’) has a significant effect on resistance to the stressful condition and the ‘Atabaki’ cultivar was a more tolerant cultivar under stress treatment.

**Figure 2:**
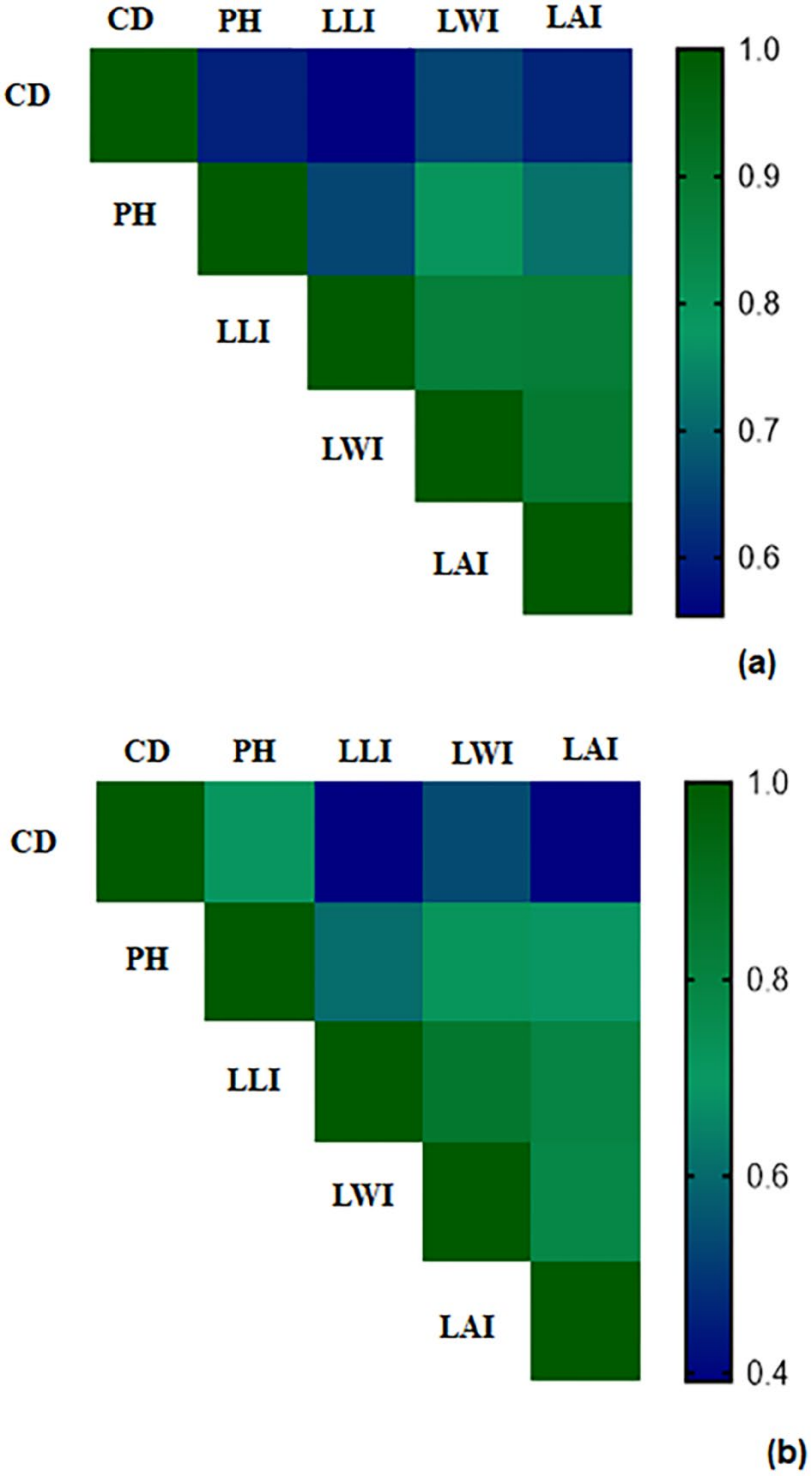
Pearson correlation analysis of morphological traits of (**a**) ‘Atabaki’ and (**b**) ‘Rabab’ in response to exogenous GABA application and drought and salinity stress. *PH* plant height, *CD* crown diameter, *LLI* leaf length index, *LWI* leaf width index, *LAI* leaf area index.

Noticeably, the growth of both pomegranate plants treated with exogenous GABA was effectively recovered under drought and salt stress, also GABA increased the growth traits of plants in non-stressed conditions. In stressful conditions, with increasing the concentration of GABA treatment (up to 40 mM), water scarcity exposed plants had higher growth traits than salinity alone and drought-salinity exposed plants (Table S1). Moreover, the maximum PH was observed through the application of 40 mM of GABA treatment. Similarly, LAI, LLI, LWI, and CD were enhanced significantly by increasing the application of GABA treatment in both cultivars at all experimental periods (Table S1). The Pearson^’^s coefficients of correlation among all the morphological traits were quantified. The positive significant correlations were detected between morphological traits in two cultivars (Fig. 2a,b). The highest linear correlation in the ‘Atabaki’ cultivar was found between LWI and LAI and in the ‘Rabab’ cultivar was obtained between LWI and LLI with values of 0.82 and 0.77. In contrast, the lowest correlation was calculated between CD and LLI in ‘Atabaki’ (r = 0.35) and ‘Rabab’ (r = 0.16) cultivars (Fig. 2a,b).

### Evaluation of MLP and RBF model

**Figure 3:**
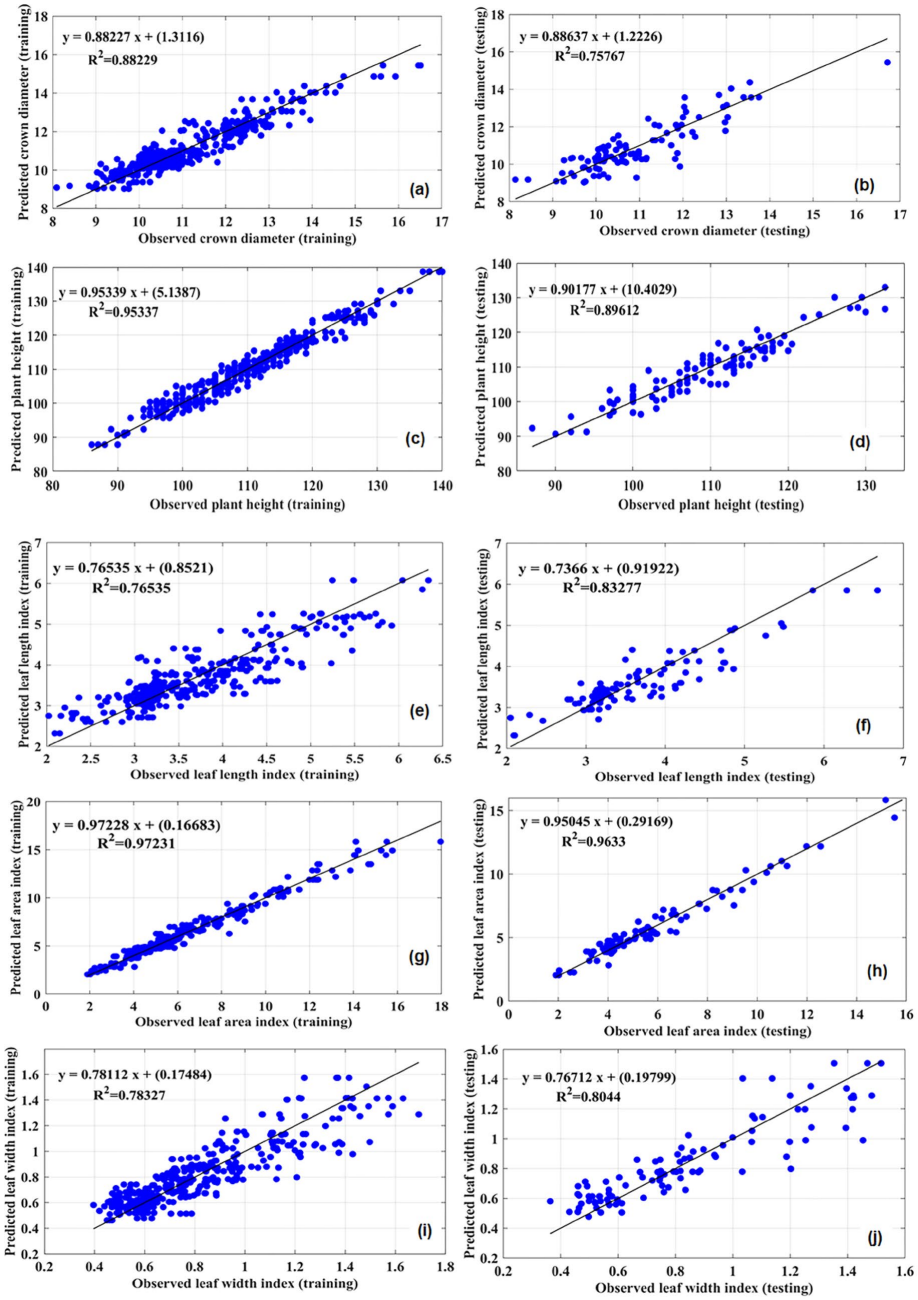
The scatter plot of observed values vs. predicted values of (**a**,**b**) crown diameter (Training) and (Testing), (**c**,**d**) plant height (Training) and (Testing), (**e**,**f**) leaf length index (Training) and (Testing), (**g**,**h**) leaf width index (Training) and (Testing), (**i**,**j**) leaf area index (Training) and (Testing) obtained by multilayer perceptron (MLP) model.

In this study, MLP and RBF models were used to predict pomegranate morphological parameters based on different GABA concentrations during salinity-drought stress. The performances of the MLP and RBF models are presented in Table 1. The accuracy of both models was evaluated by MBE and RMSE, and the results show that both models had high accuracy. However, among the two ANN algorithms tested, in most morphological parameters, the maximum value of R^2^ and the lowest value of MBE and RMSE were obtained in the MLP model. In this regard, in LLI and LWI parameters, although RBF had higher R^2^ values than MLP, the MBE and RMSE values were lower in MLP (Table 1). In both the training and testing processes of the model, the high R^2^ and the low RMSE and MBE values between the experimental (observed) and output (predicted) values represent the good fit correlation and good performance of the model for the investigated parameters. The plots of predicted stress against observed stress in the MLP model are shown in Fig. 3a–j.

**Table 1:** Comparison statistics of multilayer perceptron (MLP) and radial basis function (RBF) for various morphological traits of pomegranate.

Model	Subset	Criterion	CD	PH	LLI	LWI	LAI
MLP	Training	R^2^	0.88	0.95	0.76	0.79	0.97
		RMSE	0.46	2.1	0.30	0.11	0.47
		MBE	− 2.77	− 0.0001	− 6.33	0.0001	− 5.57
	Testing	R^2^	0.76	0.89	0.83	0.8	0.96
		RMSE	0.66	3.04	0.35	0.13	0.5
		MBE	− 0.02	− 0.34	− 0.04	0.0003	0.006
RBF	Training	R^2^	0.71	0.83	0.84	0.83	0.87
		RMSE	0.7	3.82	0.31	0.11	0.98
		MBE	− 8.99	0.0002	− 1.9	8.83	0.0001
	Testing	R^2^	0.74	0.86	0.74	0.84	0.85
		RMSE	0.8	4.16	0.38	0.13	1.08
		MBE	0.22	0.3	0.0001	0.01	0.07

*PH* plant height, *CD* crown diameter, *LLI* leaf length index, *LWI* leaf width index, *LAI* leaf area index, *R*^*2*^ coefficient of determination, *RMSE* root mean square error, *MBE* mean bias error.

### Sensitivity analysis of the models

To determine the overall VSR, the importance of each independent variable was assessed overall 512 data lines (training and testing). The results of sensitivity analysis for the model output variables (PH, CD, LLI, LWI, and LAI) with respect to input variables are summarized in Table 2. According to sensitivity analysis, a higher VSR value represents the more important input variable. Hence, using VSR values, the input variables can be ranked in terms of their importance of effect on outputs. Sensitivity analysis indicated that CD, PH, LWI, and LAI were more sensitive to pomegranate cultivars followed by GABA concentrations, drought and salinity stress, and days of treatment, respectively (Table 2). Moreover, LLI was more sensitive to pomegranate cultivars followed by GABA concentrations, days of treatment, and drought and salinity stress (Table 2). Therefore, plant cultivar was the important factor that can affect morphological traits.

**Table 2:** The sensitivity analysis to rank the importance of each input for (PH) plant height, (CD) crown diameter, (LLI) leaf length index, (LWI) leaf width index and (LAI) leaf area index of pomegranate.

Output	Item	Pomegranate cultivars	Different stresses	GABA concentrations	Days of treatment
CD	VSR	1.44	1.74	1.62	2.2
	Rank	1	3	2	4
PH	VSR	1.69	2.47	2.19	3.65
	Rank	1	3	2	4
LLI	VSR	1.11	1.57	1.37	1.56
	Rank	1	4	2	3
LWI	VSR	1.11	1.39	1.33	1.85
	Rank	1	3	2	4
LAI	VSR	2.74	3.9	3.23	4.56
	Rank	1	3	2	4

*VSE* variable sensitivity error, *VSR* variable sensitivity ratio.

### Optimization of the MLP model via NSGA-II algorithm

NSGA-II as a multi-objective optimization algorithm was linked to the MLP (the best model of this study based on accuracy) to predict and estimate the optimal values of the GABA concentrations, pomegranate cultivars, drought and salinity stress, and days of treatment to find the best morphological traits. The results of optimizations via MLP-NSGA-II are shown in Table 3. The MLP-NSGA-II analysis showed that treatment with 10.57 mM of GABA on the ‘Atabaki’ cultivar under control (non-stress) conditions after 20.80 days can be achieved optimal values of 18.42 cm, 151.82 cm, 5.67 cm, 1.76 cm, and 13.82 cm, respectively, for CD, PH, LLI, LWI and LAI (Table 3).

**Table 3:** Optimizing the concentration of GABA, days of treatment, pomegranate cultivars and salinity-drought stress according to the MLP-NSGA-II for obtaining the best pomegranate morphological traits.

Input variables								
Pomegranate cultivars	Different stresses	GABA concentrations	Days of treatment	Predicted CD	Predicted PH	Predicted LLI	Predicted LWI	Predicted LAI
Atabaki	Control	10.57	20.8	18.42	151.82	5.67	1.76	13.82

*PH* plant height, *CD* crown diameter, *LLI* leaf length index, *LWI* leaf width index, *LAI* leaf area index.

## Discussion

Morphological traits are the most important indicators that can be influenced by drought and salinity stress. The mentioned stresses reduce leaf growth and leaf water potential, induce the leaf stomatal closure, and then limit the rate of photosynthesis per unit leaf area^6–45^. Likewise, they greatly suppress cell division and growth due to the low turgor pressure. Also, the smaller leaves, decrease in plant height with a decline in the cell enlargement, reduction in diameter of the main stem, and a smaller root system are the negative consequences of drought and salinity^6–46^. The main objective of the current study is to investigate the effect of exogenous GABA for predicting the morphological traits of pomegranate plants (e.g., CD, PH, LLI, LWI, and LAI) under drought and salinity stress. To assessment of the pomegranate leaf dimensions (LLI, LWI, and LAI), image processing as an automatic method was used. The application of image processing as a fast and accurate method for measuring the morphological traits of the plant, especially in the measurement of leaf dimensions that cannot be measured via conventional measurement methods, such as area, length and width has been previously reported in several studies methods^17,36–39,43^. In order to application of GABA treatment to suppress the drought and salinity stress, the approach of a machine vision method can be the best option to manage stresses in large-scale areas as well as to detect the changes in morphological plant responses over time. This approach clearly showed that the stress treatments reduced leaf parameters. In the current study, the primary result elucidated that drought and salinity had adverse effects on morphological traits in pomegranate. But it was observed that the negative effect of drought and salinity on PH, CD, and leaf parameters was mitigated when exogenous GABA was applied to pomegranate plants. Also, in control (non-stress) conditions, the GABA-treated pomegranate plants in comparison with untreated plants demonstrated higher plant growth parameters. The growth parameters improvement by GABA application during stress could be due to stimulation of cell elongation and division, and/or maintenance of metabolic balance in plant tissues. Similarly, improving the morphological features by GABA application has been observed in several plant crops under abiotic stresses such as maize (*Zea mays*)^47^, *Vicia faba*^15^, *Phaseolus vulgaris*^48^, and sunflower (*Helianthus annuus* L.)^49^. For instance, Abdel Razik et al*.*^49^ reported that the effect of exogenous GABA under drought and heat stress lead to improve growth parameters such as plant height in sunflower.

In most cases, classical statistical methods have been commonly used to analyze morphological traits under stressful conditions^15–48^. The classical statistical analyses (e.g., ANOVA, regression, t-tests, and correlation analysis) are scheduled for small datasets and linear datasets^18–50^. Since the morphological parameters of pomegranate are multivariable processes and are affected by different factors; however, classical statistics cannot be the best option. On the other hand, the complex and multi-factorial nature of biological studies makes these studies difficult to interpret. Powerful computational tools can help biological researchers to improve the efficiency of their techniques and to use better tools in complex biological processes. ANNs, Partial least squares regression (PLSR), random forest (RF), and support vector machines (SVM) using complex mathematical functions have a high potential to analyze non-deterministic, nonparametric, and nonlinear datasets of plant studies^28–50^. Among these, the ANN technique has sufficient efficiency for both qualitative and quantitative analysis in pattern recognition in the appearance of complication conditions, when the analysis is frequently subjected to many of the noisy and imprecise input patterns^51^. Thus, to discover the relationship among the input patterns and their targets, ANN can vie with biological neurons^51,52^. Modeling and predicting biological studies such as the effects of abiotic and biotic stresses in plants through ANNs can be recognized as the influencing factors. Hence, we compared the prediction accuracy of MLP and RBF on morphological changes in the presence of exogenous GABA treatment and drought and salinity stress. Based on the results of this study, the MLP model has outperformed the RBF model, and the statistical error indices were within an acceptable range. The efficiency of MLP to predict the multi-factorial and unpredictable plant science datasets has been proved in several studies^17–24,50–53^. In order to find the best morphological traits, attaining the optimization of experimental variables is very important. Therefore, after diagnosing the MLP model as the best model based on the highest accuracy, the NSGA-II algorithm was linked to the MLP. The results of MLP-NSGA-II showed the predicted optimal input variables included 10.57 mM of GABA treatment on the ‘Atabaki’ cultivar under control (non-stress) conditions after 20.80 days to maximize morphological traits. The result of the MLP-NSGA-II algorithm was confirmed with the sensitivity analysis ranking, as the ‘Atabaki’ cultivar was the most important cultivar for morphological traits. In other words, the ‘Atabaki’ cultivar was more resistant to drought and salinity stress than the ‘Rabab’ cultivar. The results of this research demonstrated that the applied methodology is an efficient approach for estimating the effect of GABA concentrations and drought and salinity stress on the morphological traits in pomegranate. Although there is one investigation that exists for use of comparative analysis of ML algorithms and optimization algorithms in the field of plant morphophysiological responses to stress^19^, several other studies have previously reported the successful use of the NSGA-II algorithm for predicting optimized solutions in plant tissue culture^54^ and remote sensing^55^. In line with our findings, Jafari and Shahsavar^19^ reported that ANN-NSGA-II had good performance in modeling and optimizing morphological responses to drought stress in citrus plants. According to the author’s knowledge, this was the first time that ANN-NSGA-II was applied for modeling and optimization of pomegranate morphological traits under drought and salinity stress. The results of the advanced computational tool in this study can open a new window about the effect of exogenous GABA under drought and salinity stress in different plants.

## Conclusion

This study was conducted with the aim of predicting and understanding the specific effect of GABA treatment and drought and salinity stress on pomegranate morphological traits by providing the two most well-known predictive models including MLP and RBF, for the first time. The results of comparison ANN models demonstrated that MLP was more prediction accuracy than RBF. The MLP model was introduced with the NSGA-II algorithm to achieve the optimal morphological traits by the optimal input variables. The following conclusions have been obtained based on the results of the current study:

- Drought and salt stress, especially the combination of two stresses had negative impacts on morphological traits in pomegranate cultivars.
- The results obtained from the current study confirm the beneficial effects of exogenous GABA on morphological traits in response to drought, salinity, and as well as non-stress conditions. Therefore, the use of GABA seems to be a promising method to reduce the adverse effects of drought stress.
- Based on the result of sensitivity analysis, the pomegranate cultivar was a very important parameter in comparison with other parameters that affect on morphological responses. Hence, the ‘Atabaki’ cultivar was introduced as a stress-resistant cultivar.
- MLP-NSGA-II was an efficacious algorithm to predict simultaneously the best morphological responses to multivariable parameters studied.
- This utilized advanced ML modeling techniques can be applied as an alternative approach to traditional statistics for optimization and predictions of morphophysiological responses of different plants against stressful conditions in future studies.

## Supplementary Information

Supplementary Table S1.

## Data Availability

The authors confirm that the datasets analyzed during the current study are available from the corresponding author on request.
