# Supplementary material for: Artificial neural network-based model to predict the effect of γ-aminobutyric acid on salinity and drought responsive morphological traits in pomegranate
Source: Sci Rep. 2022 Oct 5;12:16662. doi: 10.1038/s41598-022-21129-z (PMC9534893; doi:10.1038/s41598-022-21129-z)
Supplement: Supplementary file 1 — Supplementary Table S1. [file 41598_2022_21129_MOESM1_ESM.docx]

**Artificial neural network-based model to predict the effect of γ-aminobutyric acid on salinity and drought responsive morphological traits in pomegranate**

**Saeedeh Zarbakhsh, Ali Reza Shahsavar**

**Table S1.** The effect of GABA concentration, days after applying treatments, pomegranate species and salinity-drought stress on morphological parameters of pomegranate.

| **Pomegranate** | | **Different** | | **GABA** | | **Days of** | **CD** | | **PH** | | **LLI** | | | **LWI** | **LAI** | |
| --- | --- | --- | --- | --- | --- | --- | --- | --- | --- | --- | --- | --- | --- | --- | --- | --- |
| **cultivars** | | **stresses** | | **concentrations** | | **treatment** |  |  |  |  |  |  |  |  |  |  |
| **‘Atabaki’** | | C | | 0 | | 0 | 10.26±0.409 | | 99.50±2.082 | | 3.19±0.103 | | | 0.54±0.030 | 4.09±0.166 | |
|  | | C | | 0 | | 14 | 10.25±0.444 | | 107.25±3.594 | | 4.09±0.121 | | | 0.73±0.028 | 4.13±0.125 | |
|  | | C | | 0 | | 30 | 10.74±0.385 | | 115.37±3.092 | | 3.62±0.576 | | | 0.94±0.094 | 7.97±0.563 | |
|  | | C | | 0 | | 45 | 11.29±0.753 | | 123.75±3.50 | | 4.11±0.154 | | | 1.16±0.077 | 8.61±0.322 | |
|  | | C | | 10 | | 0 | 10.28±0.143 | | 101.00±1.826 | | 3.17±0.046 | | | 0.52±0.059 | 4.01±0.061 | |
|  | | C | | 10 | | 14 | 10.97±0.268 | | 110.5±1.291 | | 4.33±0.244 | | | 0.90±0.131 | 5.89±0.450 | |
|  | | C | | 10 | | 30 | 12.82±0.302 | | 118.25±1.708 | | 4.28±0.225 | | | 1.10±0.067 | 9.21±0.323 | |
|  | | C | | 10 | | 45 | 16.83±0.947 | | 125.12±2.839 | | 5.22±0.357 | | | 1.35±0.077 | 13.57±1.345 | |
|  | | C | | 20 | | 0 | 10.70±0.845 | | 103.00±3.559 | | 3.15±0.047 | | | 0.57±0.068 | 3.93±0.063 | |
|  | | C | | 20 | | 14 | 10.85±0.600 | | 110.75±2.986 | | 4.71±0.075 | | | 0.88±0.101 | 6.78±0.325 | |
|  | | C | | 20 | | 30 | 11.94±0.909 | | 123.62±4.230 | | 5.78±0.500 | | | 1.40±0.145 | 15.69±1.630 | |
|  | | C | | 20 | | 45 | 12.93±0.614 | | 126.87±3.424 | | 6.27±0.333 | | | 1.46±0.071 | 14.87±0.781 | |
|  | | C | | 40 | | 0 | 10.36±0.533 | | 101.00±4.320 | | 3.10±0.079 | | | 0.53±0.061 | 4.05±0.088 | |
|  | | C | | 40 | | 14 | 11.93±0.497 | | 112.00±1.826 | | 4.52±0.075 | | | 1.21±0.051 | 10.07±0.032 | |
|  | | C | | 40 | | 30 | 12.32±0.290 | | 119.12±4.008 | | 5.40±0.275 | | | 1.20±0.211 | 12.84±0.779 | |
|  | | C | | 40 | | 45 | 13.83±0.822 | | 127.62±1.702 | | 4.75±0.170 | | | 1.12±0.063 | 10.55±0.242 | |
|  | | D | | 0 | | 0 | 10.38±0.296 | | 94.75±3.202 | | 3.11±0.136 | | | 0.44±0.077 | 4.00±0.199 | |
|  | | D | | 0 | | 14 | 9.83±0.328 | | 105.5±1.732 | | 3.20±0.029 | | | 0.60±0.020 | 3.91±0.056 | |
|  | | D | | 0 | | 30 | 10.48±0.644 | | 110.5±0.707 | | 3.83±0.208 | | | 0.68±0.061 | 4.76±0.799 | |
|  | | D | | 0 | | 45 | 11.93±2.003 | | 114.62±2.983 | | 3.69±0.219 | | | 0.72±0.054 | 5.15±0.205 | |
|  | | D | | 10 | | 0 | 10.31±0.027 | | 97.50±2.516 | | 3.21±0.047 | | | 0.52±0.037 | 3.77±0.390 | |
|  | | D | | 10 | | 14 | 10.48±0.415 | | 106.50±2.380 | | 4.10±0.225 | | | 0.73±0.032 | 4.51±0.018 | |
|  | | D | | 10 | | 30 | 14.97±2.073 | | 111.25±2.754 | | 5.03±0.618 | | | 1.13±0.063 | 14.78±0.832 | |
|  | | D | | 10 | | 45 | 15.92±1.391 | | 117.38±0.75 | | 5.17±0.247 | | | 1.44±0.134 | 10.83±0.178 | |
|  | | D | | 20 | | 0 | 12.40±0.857 | | 100.00±2.160 | | 3.19±0.040 | | | 0.54±0.062 | 3.97±0.119 | |
|  | | D | | 20 | | 14 | 11.44±0.803 | | 109.50±1.00 | | 4.49±0.080 | | | 0.82±0.069 | 5.29±0.264 | |
|  | | D | | 20 | | 30 | 12.08±0.766 | | 119.12±1.031 | | 3.51±0.081 | | | 1.06±0.099 | 10.29±0.618 | |
|  | | D | | 20 | | 45 | 13.47±0.434 | | 120.87±3.172 | | 5.44±0.295 | | | 1.54±0.118 | 10.85±0.534 | |
|  | | D | | 40 | | 0 | 10.31±0.690 | | 99.75±3.304 | | 3.12±0.067 | | | 0.52±0.082 | 3.57±0.530 | |
|  | | D | | 40 | | 14 | 8.52±0.596 | | 105.00±2.160 | | 4.02±0.032 | | | 0.90±0.041 | 5.97±0.052 | |
|  | | D | | 40 | | 30 | 9.99±0.271 | | 112.5±2.645 | | 4.90±0.549 | | | 1.18±0.077 | 10.85±0.304 | |
|  | | D | | 40 | | 45 | 12.71±0.592 | | 119.5±2.887 | | 5.32±0.251 | | | 1.40±0.021 | 12.18±0.924 | |
|  | | S | | 0 | | 0 | 10.51±0.564 | | 103.25±3.775 | | 3.10±0.079 | | | 0.55±0.091 | 3.98±0.136 | |
|  | | S | | 0 | | 14 | 8.45±0.289 | | 108.25±2.754 | | 2.69±0.217 | | | 0.50±0.026 | 3.46±0.149 | |
|  | | S | | 0 | | 30 | 10.18±0.347 | | 108.75±2.630 | | 2.87±0.447 | | | 0.51±0.040 | 4.31±0.173 | |
|  | | S | | 0 | | 45 | 10.81±0.902 | | 111.00±2.708 | | 2.79±0.172 | | | 0.85±0.022 | 4.64±0.491 | |
|  | | S | | 10 | | 0 | 10.59±0.089 | | 105.5±1.732 | | 3.17±0.022 | | | 0.56±0.061 | 4.00±0.149 | |
|  | | S | | 10 | | 14 | 10.84±0.657 | | 91.00±3.559 | | 3.33±0.271 | | | 0.72±0.030 | 5.46±0.099 | |
|  | | S | | 10 | | 30 | 12.17±0.795 | | 107.75±2.986 | | 3.77±0.122 | | | 0.90±0.047 | 5.64±0.440 | |
|  | | S | | 10 | | 45 | 12.20±0.675 | | 116.37±1.25 | | 4.26±0.022 | | | 1.33±0.108 | 6.78±1.200 | |
|  | | S | | 20 | | 0 | 10.64±0.403 | | 105.75±0.957 | | 3.16±0.144 | | | 0.53±0.073 | 4.04±0.148 | |
|  | | S | | 20 | | 14 | 9.90±0.829 | | 107.25±0.50 | | 4.13±0.088 | | | 0.70±0.066 | 5.60±0.084 | |
|  | | S | | 20 | | 30 | 11.00±0.740 | | 111.63±1.973 | | 3.56±0.026 | | | 0.70±0.008 | 5.29±0.302 | |
|  | | S | | 20 | | 45 | 12.37±1.524 | | 115.13±0.629 | | 3.70±0.661 | | | 1.06±0.126 | 6.38±0.429 | |
|  | | S | | 40 | | 0 | 10.33±0.634 | | 105.88±1.031 | | 3.21±0.049 | | | 0.57±0.062 | 4.01±0.036 | |
|  | | S | | 40 | | 14 | 12.79±1.256 | | 114.00±2.828 | | 3.18±0.116 | | | 0.64±0.037 | 4.46±0.301 | |
|  | | S | | 40 | | 30 | 12.81±0.825 | | 116.00±3.342 | | 3.36±0.109 | | | 0.77±0.064 | 5.71±0.126 | |
|  | | S | | 40 | | 45 | 15.05±2.851 | | 118.75±0.866 | | 3.55±0.224 | | | 1.06±0.129 | 6.86±0.388 | |
|  | | D×S | | 0 | | 0 | 10.78±0.351 | | 98.50±3.416 | | 3.12±0.056 | | | 0.56±0.059 | 3.68±0.587 | |
|  | | D×S | | 0 | | 14 | 9.86±0.258 | | 105.37±2.136 | | 2.62±0.187 | | | 0.56±0.047 | 3.04±0.091 | |
|  | | D×S | | 0 | | 30 | 10.00±0.387 | | 105.75±1.893 | | 2.85±0.120 | | | 0.52±0.051 | 5.93±0.552 | |
|  | | D×S | | 0 | | 45 | 10.95±0.404 | | 107.00±1.633 | | 2.41±0.081 | | | 0.49±0.066 | 2.70±0.173 | |
|  | | D×S | | 10 | | 0 | 10.23±0.256 | | 99.25±3.304 | | 3.09±0.063 | | | 0.63±0.028 | 3.95±0.178 | |
|  | | D×S | | 10 | | 14 | 9.81±0.109 | | 106.5±2.646 | | 3.09±0.132 | | | 0.74±0.020 | 4.66±0.272 | |
|  | | D×S | | 10 | | 30 | 10.02±0.180 | | 112.25±2.255 | | 3.78±0.322 | | | 0.85±0.031 | 7.93±0.974 | |
|  | | D×S | | 10 | | 45 | 10.48±0.582 | | 115.25±1.658 | | 2.75±0.149 | | | 0.64±0.013 | 3.67±0.082 | |
|  | | D×S | | 20 | | 0 | 10.65±0.430 | | 97.25±2.101 | | 3.13±0.127 | | | 0.59±0.026 | 3.97±0.041 | |
|  | | D×S | | 20 | | 14 | 9.78±0.591 | | 103.37±3.944 | | 3.55±0.048 | | | 0.70±0.078 | 5.78±0.425 | |
|  | | D×S | | 20 | | 30 | 9.67±0.752 | | 111.5±3.188 | | 3.83±0.687 | | | 0.85±0.096 | 8.14±1.061 | |
|  | | D×S | | 20 | | 45 | 10.75±0.483 | | 116.00±2.708 | | 3.44±0.100 | | | 0.78±0.059 | 5.50±0.503 | |
|  | | D×S | | 40 | | 0 | 11.26±0.646 | | 100.75±2.986 | | 3.17±0.017 | | | 0.56±0.049 | 3.98±0.084 | |
|  | | D×S | | 40 | | 14 | 10.83±0.170 | | 103.87±3.705 | | 3.54±0.356 | | | 0.83±0.162 | 7.70±0.083 | |
|  | | D×S | | 40 | | 30 | 10.54±0.387 | | 111.75±3.304 | | 3.80±0.172 | | | 1.09±0.158 | 10.13±0.697 | |
|  | | D×S | | 40 | | 45 | 12.66±0.502 | | 117.13±0.854 | | 3.60±0.005 | | | 1.01±0.119 | 6.52±0.274 | |
| **‘Rabab’** | | C | | 0 | | 0 | 11.05±0.891 | | 101.63±1.377 | | 3.14±0.092 | | | 0.57±0.058 | 4.16±0.029 | |
|  | | C | | 0 | | 14 | 11.40±0.711 | | 113.88±0.854 | | 3.16±0.041 | | | 0.56±0.037 | 5.00±0.058 | |
|  | | C | | 0 | | 30 | 11.92±0.604 | | 114.88±0.854 | | 3.14±0.037 | | | 0.76±0.066 | 5.69±0.077 | |
|  | | C | | 0 | | 45 | 12.71±0.835 | | 117.25±1.50 | | 4.20±0.089 | | | 0.95±0.011 | 6.71±0.268 | |
|  | | C | | 10 | | 0 | 10.63±0.137 | | 106.00±2.582 | | 3.11±0.048 | | | 0.58±0.058 | 4.17±0.029 | |
|  | | C | | 10 | | 14 | 11.71±0.721 | | 114.25±0.957 | | 3.55±0.167 | | | 0.76±0.101 | 5.59±0.225 | |
|  | | C | | 10 | | 30 | 13.02±0.899 | | 125.5±1.732 | | 4.07±0.235 | | | 0.91±0.051 | 7.25±0.490 | |
|  | | C | | 10 | | 45 | 13.85±1.039 | | 132.87±1.887 | | 4.88±0.294 | | | 1.37±0.126 | 8.84±0.631 | |
|  | | C | | 20 | | 0 | 10.73±0.497 | | 97.50±3.109 | | 3.19±0.017 | | | 0.60±0.028 | 4.16±0.008 | |
|  | | C | | 20 | | 14 | 10.99±0.219 | | 106.37±1.887 | | 3.69±0.218 | | | 0.77±0.071 | 5.47±0.098 | |
|  | | C | | 20 | | 30 | 11.91±0.464 | | 125.25±1.936 | | 3.35±0.323 | | | 0.79±0.016 | 8.92±0.001 | |
|  | | C | | 20 | | 45 | 14.22±0.257 | | 129.12±2.689 | | 4.86±0.042 | | | 1.30±0.085 | 8.43±0.176 | |
|  | | C | | 40 | | 0 | 10.16±0.130 | | 99.75±3.096 | | 3.12±0.215 | | | 0.53±0.055 | 4.17±0.022 | |
|  | | C | | 40 | | 14 | 10.24±0.157 | | 103.5±1.472 | | 3.55±0.272 | | | 0.85±0.081 | 5.87±0.039 | |
|  | | C | | 40 | | 30 | 11.54±0.499 | | 128.12±3.568 | | 4.03±0.213 | | | 1.15±0.111 | 9.44±0.795 | |
|  | | C | | 40 | | 45 | 12.05±0.482 | | 138.62±1.377 | | 5.59±0.328 | | | 1.45±0.173 | 12.21±0.171 | |
|  | D | | 0 | | 0 | | | 10.75±0.134 | | 100.25±3.096 | | 3.15±0.049 | 0.56±0.052 | | 4.11±0.007 |  |
|  | D | | 0 | | 14 | | | 9.39±0.254 | | 101.50±3.786 | | 3.09±0.047 | 0.56±0.031 | | 4.89±0.046 |  |
|  | D | | 0 | | 30 | | | 10.55±0.336 | | 111.37±2.287 | | 3.05±0.022 | 0.76±0.088 | | 5.11±0.038 |  |
|  | D | | 0 | | 45 | | | 11.41±0.938 | | 112.13±2.175 | | 3.24±0.083 | 0.91±0.048 | | 5.25±0.093 |  |
|  | D | | 10 | | 0 | | | 10.44±0.213 | | 96.25±1.708 | | 3.21±0.096 | 0.54±0.043 | | 4.17±0.042 |  |
|  | D | | 10 | | 14 | | | 9.39±0.122 | | 99.25±1.708 | | 3.57±0.087 | 0.74±0.013 | | 5.18±0.037 |  |
|  | D | | 10 | | 30 | | | 9.69±0.101 | | 109.5±2.646 | | 4.84±0.096 | 1.06±0.104 | | 6.30±0.161 |  |
|  | D | | 10 | | 45 | | | 12.07±0.156 | | 115.00±2.160 | | 4.86±0.295 | 1.29±0.125 | | 5.59±0.626 |  |
|  | D | | 20 | | 0 | | | 10.76±0.869 | | 108.50±1.291 | | 3.13±0.077 | 0.60±0.068 | | 4.04±0.176 |  |
|  | D | | 20 | | 14 | | | 9.66±0.382 | | 109.50±3.109 | | 3.84±0.039 | 0.78±0.026 | | 5.20±0.034 |  |
|  | D | | 20 | | 30 | | | 10.57±0.403 | | 114.00±2.449 | | 4.39±0.043 | 0.75±0.052 | | 7.66±0.340 |  |
|  | D | | 20 | | 45 | | | 11.53±0.923 | | 119.25±2.217 | | 4.71±0.535 | 1.00±0.047 | | 6.82±0.734 |  |
|  | D | | 40 | | 0 | | | 10.69±0.270 | | 99.00±1.826 | | 3.14±0.072 | 0.58±0.088 | | 4.08±0.072 |  |
|  | D | | 40 | | 14 | | | 10.31±0.258 | | 104.25±2.986 | | 3.78±0.101 | 0.84±0.044 | | 5.06±0.518 |  |
|  | D | | 40 | | 30 | | | 12.43±0.652 | | 124.75±1.936 | | 4.05±0.039 | 0.97±0.111 | | 8.85±0.101 |  |
|  | D | | 40 | | 45 | | | 13.14±1.477 | | 128.88±1.931 | | 4.54±0.195 | 1.38±0.098 | | 8.86±0.167 |  |
|  | S | | 0 | | 0 | | | 10.63±0.253 | | 101.75±2.363 | | 3.13±0.045 | 0.57±0.052 | | 4.13±0.013 |  |
|  | S | | 0 | | 14 | | | 10.54±0.487 | | 110.5±3.873 | | 2.11±0.027 | 0.46±0.009 | | 1.96±0.070 |  |
|  | S | | 0 | | 30 | | | 10.56±0.488 | | 116.63±2.213 | | 3.02±0.101 | 0.55±0.105 | | 2.91±0.577 |  |
|  | S | | 0 | | 45 | | | 12.67±1.071 | | 118.13±2.213 | | 2.13±0.126 | 0.50±0.042 | | 2.40±0.196 |  |
|  | S | | 10 | | 0 | | | 10.67±0.255 | | 98.25±3.227 | | 3.08±0.129 | 0.53±0.045 | | 4.16±0.057 |  |
|  | S | | 10 | | 14 | | | 11.29±0.228 | | 110.00±2.944 | | 3.21±0.086 | 0.66±0.044 | | 3.26±0.172 |  |
|  | S | | 10 | | 30 | | | 11.47±0.694 | | 116.00±1.826 | | 3.26±0.151 | 0.75±0.048 | | 4.06±0.164 |  |
|  | S | | 10 | | 45 | | | 13.98±1.301 | | 117.63±2.136 | | 3.08±0.174 | 0.75±0.062 | | 4.03±0.109 |  |
|  | S | | 20 | | 0 | | | 10.33±0.414 | | 102.5±2.887 | | 3.17±0.105 | 0.56±0.056 | | 4.11±0.094 |  |
|  | S | | 20 | | 14 | | | 9.71±0.359 | | 109.75±2.102 | | 3.30±0.207 | 0.69±0.087 | | 3.68±0.260 |  |
|  | S | | 20 | | 30 | | | 10.00±0.811 | | 116.13±2.323 | | 3.30±0.046 | 0.79±0.047 | | 5.20±0.570 |  |
|  | S | | 20 | | 45 | | | 12.02±1.407 | | 117.88±1.548 | | 3.28±0.100 | 0.81±0.085 | | 4.62±0.153 |  |
|  | S | | 40 | | 0 | | | 10.05±0.389 | | 98.88±2.657 | | 3.14±0.055 | 0.53±0.062 | | 4.16±0.031 |  |
|  | S | | 40 | | 14 | | | 10.14±1.296 | | 109.75±2.217 | | 3.93±0.224 | 0.97±0.115 | | 6.23±1.578 |  |
|  | S | | 40 | | 30 | | | 10.01±0.575 | | 117.5±1.291 | | 3.92±0.053 | 0.81±0.021 | | 6.70±0.350 |  |
|  | S | | 40 | | 45 | | | 11.72±0.733 | | 118.63±1.377 | | 3.63±0.288 | 0.83±0.047 | | 5.49±0.056 |  |
|  | D×S | | 0 | | 0 | | | 10.91±0.632 | | 87.75±1.708 | | 3.14±0.037 | 0.57±0.056 | | 4.09±0.031 |  |
|  | D×S | | 0 | | 14 | | | 9.39±0.187 | | 90.50±0.577 | | 2.30±0.023 | 0.46±0.029 | | 4.35±0.125 |  |
|  | D×S | | 0 | | 30 | | | 9.63±0.387 | | 92.13±1.315 | | 2.80±0.217 | 0.72±0.067 | | 5.30±0.061 |  |
|  | D×S | | 0 | | 45 | | | 10.78±0.587 | | 97.63±2.136 | | 3.07±0.091 | 0.75±0.035 | | 2.33±0.331 |  |
|  | D×S | | 10 | | 0 | | | 10.71±0.568 | | 102.5±3.512 | | 3.11±0.057 | 0.58±0.013 | | 4.11±0.092 |  |
|  | D×S | | 10 | | 14 | | | 9.54±0.343 | | 109.38±2.358 | | 2.89±0.149 | 0.52±0.037 | | 5.64±0.094 |  |
|  | D×S | | 10 | | 30 | | | 9.78±0.210 | | 110.88±0.629 | | 3.31±0.285 | 1.06±0.169 | | 6.19±0.314 |  |
|  | D×S | | 10 | | 45 | | | 12.01±0.896 | | 113.63±1.493 | | 3.73±0.203 | 1.02±0.161 | | 5.48±0.060 |  |
|  | D×S | | 20 | | 0 | | | 10.51±0.310 | | 101.75±3.947 | | 3.16±0.057 | 0.57±0.032 | | 4.16±0.021 |  |
|  | D×S | | 20 | | 14 | | | 9.90±0.220 | | 105.25±4.573 | | 3.31±0.166 | 0.75±0.049 | | 5.17±0.063 |  |
|  | D×S | | 20 | | 30 | | | 11.01±0.613 | | 107.75±1.708 | | 3.65±0.188 | 0.77±0.080 | | 6.65±0.097 |  |
|  | D×S | | 20 | | 45 | | | 12.60±0.657 | | 118.00±3.764 | | 3.67±0.183 | 1.08±0.248 | | 5.36±0.210 |  |
|  | D×S | | 40 | | 0 | | | 10.34±0.279 | | 100.38±2.75 | | 3.11±0.058 | 0.60±0.021 | | 4.16±0.018 |  |
|  | D×S | | 40 | | 14 | | | 9.81±0.447 | | 104.88±2.780 | | 3.71±0.131 | 0.82±0.053 | | 4.50±0.136 |  |
|  | D×S | | 40 | | 30 | | | 10.29±0.265 | | 110.00±2.160 | | 4.02±0.417 | 0.80±0.017 | | 6.85±0.606 |  |
|  | D×S | | 40 | | 45 | | | 12.44±0.332 | | 127.25±1.708 | | 3.73±0.063 | 0.89±0.056 | | 6.19±0.282 |  |

Values in each column represent means ± SD. PH= plant height; CD= crown diameter; LLI= leaf length index ; LWI= leaf width index and LAI= leaf area index; C= control; D= drought stress; S= salinity stress; D×S= drought and salinity stress.
